# ErbB3 plays a key role in the early phase of establishment of resistance to BRAF and/or MEK inhibitors

**DOI:** 10.1186/1479-5876-13-S1-K3

**Published:** 2015-01-15

**Authors:** Luigi Fattore, Debora Malpicci, Emanuele Marra, Rosalba Camerlingo, Giuseppe Roscilli, Francesca Belleudi, Antoni Ribas, Rita Mancini, Maria Rosaria Torrisi, Luigi Aurisicchio, Paolo Antonio Ascierto, Gennaro Ciliberto

**Affiliations:** 1 Dipartimento di Medicina Clinica e Molecolare, Sapienza Università di Roma, Rome, Italy; 2 Dipartimento di Chirurgia “P. Valdoni”, Sapienza Università di Roma, Rome, Italy; 3 Dipartimento di Medicina Sperimentale e Clinica, Università degli Studi di Catanzaro “Magna Graecia”, Catanzaro, Italy; 4 Takis srl, Rome, Italy; 5 Istituto Nazionale per lo Studio e la Cura dei Tumori “Fondazione G. Pascale”, Naples, Italy; 6 Istituto Pasteur Fondazione Cenci Bolognetti, Dipartimento di Medicina Clinica e Molecolare, Sapienza Università di Roma, Rome, Italy; 7 Department of Medicine, Division of Hematology/Oncology, University of California Los Angeles (UCLA), Los Angeles, CA, USA; 8 Azienda Ospedaliera S. Andrea, Rome, Italy

## Background

A major issue in the management of cancer is the development of drug resistance. In metastatic melanoma bearing V600 mutations in the BRAF oncogene, all patients undergo disease relapse after combination therapy with BRAF and MEK inhibitors. Hence, understanding the mechanisms at the basis of development of resistance is fundamental to the discovery of new therapeutic approaches. In our group we have spent the last years to identify mechanisms of early adaptation of BRAF mutated melanoma to BRAF and or MEK inhibitors. We have recently shown that the ErbB3 receptor is involved in the activation of an early feedback survival loop upon cell exposure to BRAF and/or MEK inhibitors. Upregulation of pErbB3, due to enhanced production of its ligand neuregulin-1 (HRG), causes increased AKT phosphorylation and cell survival. Furthermore, we demonstrated that activation of the ErbB3/AKT axis is abrogated by co-treatment with anti-ErbB3 mAbs previously generated in our laboratory.

## Materials and methods

Eleven different melanoma cell lines bearing BRAF V600E or BRAF V600D or BRAF V600R mutations were exposed to short term or long term treatment with vemurafenib and/or trametinib and/or anti ErbB3 antibodies A3 and A4. Short term growth inhibition was measured by colony forming assays, cell cycle and apoptosis markers. Long term treatments allowed the selection of resistant clones. Western blot analysis was performed on total protein extracts using anti-ErbB3, anti-AKT and anti-ERK 1/2 antibodies. Mouse xenograft studies were carried out with M14 cells injected s.c. at the dose of 5x 10^6^ cells. Individual or combined drug treatments began when tumors reached a mean volume of 100mm^3^ and tumor growth was measured by caliper.

## Results

We show that ErbB3 undergoes a strong upregulation of its phosphorylation in the absence of external addition of neuregulin (HRG) upon exposure to vemurafenib or trametinib or both drugs in the 10 out of 11 of cell lines tested. Phospho ErbB3 activation is accompanied by strong phosphorylation of downstream AKT. Most importantly anti-ErbB3 monoclonal antibodies combination strongly enhances the ability of BRAF/MEK inhibitors to silence the oncogenic MAPK and AKT pathways. This results in potentiation of growth inhibition and of apoptosis compared to single antibody treatments. Moreover ErbB3 mAbs impair the establishment of resistance and restore drug sensitivity to vemurafenib in resistant melanoma cells. Finally anti-ErbB3 mAbs A3 and A4 combination strongly affect *“in vivo”* melanoma cell growth and reduces tumor relapse when combined with vemurafenib and tramentinib.

## Conclusions

Feedback activation of ErbB3/AKT phosphorylation is a fast and common response of melanoma cells to BRAF and/or MEK inhibitors. Here, we show for the first time that the ErbB3 receptor is a key-player also in long-time drug establishment of resistance. These data strongly underline the role of ErbB3 in the rebound of melanoma cell growth following vemurafenib/trametinib treatments and pave the way for the use of anti-ErbB3 mAbs as adjuncts to current target therapies in order to obtain a durable control of tumor growth.

